# mSphere of Influence: That’s Racist—COVID-19, Biological Determinism, and the Limits of Hypotheses

**DOI:** 10.1128/mSphere.00945-20

**Published:** 2020-09-30

**Authors:** Kishana Taylor

**Affiliations:** a Carnegie Mellon University, Pittsburgh, Pennsylvania, USA

**Keywords:** Black In Microbiology, COVID-19, SARS-CoV-2, health disparities

## Abstract

Kishana Taylor works in the field of virology. In this mSphere of Influence article, she reflects on the personal impact of “Racial health disparities and COVID-19 – caution and context” by Merlin Chowkwanyun and Adolph L. Reed, Jr. (N Engl J Med 383:201–203, 2020, https://doi.org/10.1056/NEJMp2012910) and “A hypothesis is a liability” by Itai Yanai and Martin Lercher (Genome Biol 21:231, 2020, https://doi.org/10.1186/s13059-020-02133-w) and how it became part of the mission for Black In Microbiology Week.

## COMMENTARY

Black In Microbiology Week was created following the latest in a series of police killings—Ahmaud Arbury, George Floyd, and Breonna Taylor, among others—that created an atmosphere where our contemporaries and superiors were willing to have open and honest conversations about the experiences of Black people in STEM (science, technology, engineering, and mathematics). Like the many Black in STEM discipline movements on Twitter recently (e.g., Black in Neuro and Black in Botany), we wanted to connect and celebrate Black microbiologists. Given that these moments also occurred during the severe acute respiratory syndrome coronavirus 2 (SARS-CoV-2) pandemic, we considered this an opportunity to highlight the coronavirus disease 2019 (COVID-19) disparities observed in Black, native, and other communities of color compared to white communities. For these reasons, we established the mission of Black In Microbiology Week as the following:

- Showcase the presence and accomplishments of Black microbiologists from around the globe.
- Connect Black microbiologists with one another and foster a sense of community among them.
- Provide a forum to discuss racial disparities in microbiology and its subdisciplines.
- Amplify Black scientists in all disciplines, acknowledge the contributions to their disciplines, and support the collective work of pursuing equity in academia, industry, government, and beyond.

A recent paper in *Genome Biology* (1) described the flaws of hypothesis-driven science including how it often leads to scientists missing novel discoveries and stifled creativity. I argue that hypothesis-driven data are flawed in additional ways, especially when it comes to infectious disease microbiology and health disparities research. As scientists, we seek to have neutral hypotheses. In other words, hypotheses are supposed to be free of bias and based solely on the data. For the sake of this commentary, I define bias as preconceived notions or experiences that can influence our interpretation of data. However, this aim is flawed, as even the way we think, the way we interpret data, is influenced by our experiences through life.

Oftentimes, as microbiologists, our hypotheses and experiments are informed by data, and often our biases, but not the context or history behind the data or the biases. It can be easy to dismiss the contributing context as a different discipline, but this context should inform how we conduct our research and examine our hypotheses before we even begin data collection. The field of epigenetics has taught us that on a genetic scale, humans of all races are genetically similar (2). We also know that the environment can greatly influence what, when, and how genes are activated. From a perspective that does not consider the social determinants of health on a macroscale, it may seem that race is the underlying factor between differences in disease rates. But examining the context of the data and the racial categories shows that racism (i.e., how an individual categorized into a racial group is treated), and not race, is the cause. Institutional and structural racism, racism that is embedded into the normal day to day practice and underpinnings of society, determines the access a person has to different environments, as well as how that person is treated in day to day interactions. So when we see data that say Black people have higher rates of COVID-19, we have to first consider what role institutional and structural racism have in shaping the environments in which they live and work.

A recent perspective published in the *New England Journal of Medicine*, written by Dr. Merlin Chowkwanyun and Dr. Adolph L. Reed, Jr. (3) cautioned against the slippery slope of observing racial disparities in the rates of COVID-19 cases and deaths and then relating them to biological determinism. Dr. Chowkwanyun and Dr. Reed advocate for examining the disparity data with the “explanatory context in order to avoid perpetuating harmful myths and misunderstanding that actually undermine the goal of eliminating health inequities.” This is an important point to make, as many hypotheses have already attempted to blame biological differences between racial groups to explain the disparities away. Examples include vitamin D deficiency, poor diets, and differential expression of the enzyme TMPRSS2, among others. The problem with immediately trying to find a biologic cause for health disparities is that it ignores the social aspects of disease, also known as the social determinants of health (SDOH). SDOH, as defined by the CDC, are “conditions in the places where people live, learn, work, and play that affect a wide range of health and quality-of-life risks and outcomes (4).” Factors contributing to disease that could be considered a SDOH include access to health care and quality of the care.

With Black In Microbiology Week, we want to bring awareness to how the surface level analysis of infectious disease health disparities data often leads to racist hypotheses. Ignoring the effects of institutional and personal racism on Black people’s access to health, wealth, or their ability to physically distance, we perpetuate racism and gaslight an entire community of people. Black microbiologists are often the only Black person in their lab or even department, making it tenuous, especially as early career researchers, to communicate this limitation to our colleagues and superiors. The Black In Microbiology committee wants to create a *safe* platform to discuss these flawed and racist hypotheses that other microbiologists can attend to understand our experiences and perspectives. We hope that our scientific colleagues can implement these antiracist concepts in their own labs and departments to further change the culture of scientific inquiry to effect greater change in our communities.

For microbiologists to do better, they need to: (i) ask themselves the purpose that their hypotheses serve, (ii) interrogate their own biases before forming a hypothesis, and (iii) consider alternative explanations for health disparities that include racism and SDOH. To continue to learn, hear from, and amplify Black microbiologists, consider following Black In Microbiology Week (28 September to 4 October 2020) with the #BlackInMicro hashtag and visiting the website blackinmicrobiology.org.
